# Identification and Biocontrol of *Fusarium oxysporum* Affecting Lucky Bamboo (*Dracaena sanderiana* Hort. ex. Mast.)

**DOI:** 10.3390/jof11090655

**Published:** 2025-09-04

**Authors:** Merve Şenol Kotan

**Affiliations:** Department of Agricultural Biotechnology, Faculty of Agriculture, University of Atatürk, 25240 Erzurum, Türkiye; merves@atauni.edu.tr

**Keywords:** *Dracaena sanderiana*, *Fusarium oxysporum*, *Pseudomonas chlororaphis*, *Agrobacterium radiobacter*, *Bacillus megaterium*, chitinase, biocontrol

## Abstract

Lucky bamboo is an economically crucial ornamental plant worldwide due to its durability, rapid growth capacity, and versatile uses. However, diseases caused by various fungal pathogens negatively affect bamboo production, resulting in yield losses. In the present study, fungal agents causing disease in *Dracaena sanderiana* were isolated and evaluated for their pathogenicity. The MF-1 and MF-2 isolates that showed pathogenicity were characterized morphologically and molecularly. Chitinase enzymes were partially purified from four different bacteria and biochemically characterized, and the antifungal activities of these bacteria and chitinases were evaluated. As a result of the diagnosis, both isolates were identified as *Fusarium oxysporum* with ~99% similarity. It was determined that the partially purified chitinases from *Pseudomonas chlororaphis* C-37A and *Agrobacterium radiobacter* A-16 had the highest activity with values of 9.44 and 1.02 EU/mL, respectively. Additionally, the pH and temperature values at which C-37A’s chitinase exhibited optimal activity were determined to be 8 and 30 °C, while those for A-16’s chitinase were found to be pH 4 and 40 °C. After 120 min, C-37A’s chitinase retained 50% of its activity at 90 °C, while A-16’s chitinase retained 80% of its activity at 40 °C. C-37A inhibited the growth of MF-1 and MF-2 by 83% and 75%, respectively. Additionally, the inhibition rates of A-16, *Bacillus megaterium* M-3, and KBA-10 ranged from 68% to 29%. In chitinase applications, the highest inhibition rates of 28% (MF-1) and 23% (MF-2) were obtained from C-37A chitinase. In conclusion, it was observed that bioagent bacteria provide sustainable biological effects against *F*. *oxysporium* in *D*. *Sanderiana*, and that the chitinase enzyme purified from these isolates can be used as a biocontrol agent in agriculture, as well as potentially evaluated in various industrial applications.

## 1. Introduction

Ornamental flowers are suitable decorative plants found both indoors and outdoors. Lucky bamboo (*Dracaena sanderiana* Hort. ex. Mast.) is a popular ornamental plant from the Asparagaceae family and holds a significant place in the global market [1]. Lucky bamboo is an upright, woody, evergreen perennial shrub with a slender trunk and a flexible ribbon-like shape [2]. A decorative flowering vase plant, lucky bamboo has gained popularity as an ornamental houseplant due to its attractive appearance, low cost, and ability to grow in low-light indoor conditions [3].

Lucky bamboo plants play an essential role in the decoration of both private and public spaces, such as homes, schools, offices, and shopping malls, to overcome serious air and environmental pollution problems [4,5]. Although these plants, also called lucky bamboo, are easy to care for and adapt, various diseases affect this species worldwide. Fungal diseases affecting lucky bamboo include *Fusarium* stem rot and wilt, Botrytis blight, and stem and leaf spots, while bacterial diseases include anthracnose, and *Erwinia* leaf rot, and stem spots [4,6,7]. Fungal diseases of lucky bamboo have been reported in many countries. For example, in China, Xiong et al. [8] identified *Colletotrichum gloeosporioides*; in Iran, Abedi-Tizaki et al. [9] identified *Fusarium solani*; and in Iraq, Lahuf et al. [4] identified *F. proliferatum*. In Egypt, Elshahawy et al. [10] reported *Colletotrichum* sp. Abdel-Rahman et al. [11] also reported *Rhizoctonia solani*, and Abdel-Rahman et al. [5] reported *C. gloeosporioides*, *Alternaria alternata*, and *Fusarium oxysporum*. *F. oxysporum* is one of the most destructive soil-borne pathogens in ornamental plant production. *F. oxysporum* can infect a wide range of ornamental plants during production and storage. *F. oxysporum* generally produces symptoms such as wilting, chlorosis, necrosis, premature leaf drop, browning of the vascular system, stunting, and dumping-off. The most important is vascular wilt [5,12].

Disease management can become more effective by integrating biological control options [13]. Plant diseases and pests are controlled through biocontrol applications, using Plant Growth-Promoting Bacteria (PGPB). PGPB exhibit antagonistic effects against pathogens by increasing plant systemic resistance, protecting plants against many diseases and stress conditions, and synthesizing compounds such as antibiotics, siderophores, vitamins, enzymes, and fungicides [14,15]. Chitinases hold a significant place among the enzymes used in biological control. Chitinases are hydrolytic enzymes that degrade chitin, a key polysaccharide component of fungal cell walls. By disrupting this structural barrier, chitinases disturb the integrity of fungal pathogens, leading to growth inhibition or cell degradation. This mechanism of chitinase action supports using chitinase-producing microorganisms as effective biological control agents against plant pathogens [16]. This enzyme offers effective, environmentally friendly, and sustainable biological effects, particularly against various plant pathogens that cause significant declines in crop yields worldwide [17]. In this study, fungal agents causing disease on *D. sanderiana* were isolated, and their pathogenicity was evaluated. Two *Fusarium oxysporum* isolates (MF-1 and MF-2) causing *Fusarium* wilt were identified at the morphological and molecular levels. The antifungal activities of bioagent isolates (*Pseudomonas chlororaphis* C37-A, *Bacillus megaterium* M-3, *B. megaterium* KBA-10, *Agrobacterium radiobacter* A-16) and the partially purified chitinase enzyme were evaluated. Optimum pH, temperature, and stability conditions for the enzyme were determined, and the biocontrol potential of these strains and the enzyme were investigated. This is the first study conducted on *Fusarium oxysporum*, the fungus causing *Fusarium* wilt on lucky bamboo stems in Türkiye.

## 2. Materials and Methods

### 2.1. Bioagent Bacteria, and Plants Used in the Study

*Pseudomonas chlororaphis* C37-A (MW740242), *Bacillus megaterium* M-3, *B. megaterium* KBA-10, *Agrobacterium radiobacter* A-16 bacteria were selected due to their biological control properties. Potential biocontrol bacteria were obtained from the culture collection unit in the Department of Plant Protection, Faculty of Agriculture at Ataturk University, Turkey [14,15,16]. *D. sanderiana* plants exhibiting typical wilt symptoms were obtained from local nurseries in Erzurum, Türkiye, during May 2024. This plant was used for pathogen isolation and subsequent analyses.

### 2.2. Isolation of Pathogenic Fungi

Disease-infected *D. sanderiana* specimens were obtained from ornamental flower vendors in Erzurum province. Laboratory specimens were sterilized with 70% ethyl alcohol for 3 min and then involved surface sterilization of infected plant tissues in 1% sodium hypochlorite (NaOCl) for 1–2 min. Following sterilization, the diseased sections were placed on Potato Dextrose Agar (PDA) and incubated at 28 °C for 5 days. Within 4–5 days, hyphal tips were taken from the edge of the colonies and transferred to PDA-containing Petri dishes to obtain five different pure cultures. These isolates were then transferred to PDA-containing test tubes and stored at 4 °C for future use [18].

### 2.3. Pathogenicity and Virulence Tests

Each of the five fungi obtained from *D. sanderiana* was subjected to pathogenicity and virulence tests. Five healthy lucky bamboos were used for each treatment. After washing the lucky bamboos with tap water, they were sterilized with 70% ethyl alcohol for 3 min and then involved surface sterilization of infected plant tissues in 1% sodium hypochlorite (NaOCl) for 1–2 min, and a 5 mm diameter wound was created at the apex of the plants using a sterile scalpel [18]. The concentration of pathogenic fungal isolates was adjusted to 10^6^ arthrospores/mL using a hemocytometer, and 20 µL of spore suspension was injected into the wounds [19]. The wounds were wrapped with parafilm. Sterile water was used in the control treatment. The plants were stored at 25 °C and 80% relative humidity for 15 days, with a period of 8 h of darkness and 16 h of light [11]. After 15 days, only isolates MF-1 and MF-2 from the fungal inoculated lucky bamboos showed wilt symptoms, but the control plants showed no symptoms during the entire evaluation period (15 days). Virulence of fungal isolates on lucky bamboo was determined on a scale (0–4) (0 = no disease; 1 = 1–25% slight wilting and yellowing, 2 = 26–50% pronounced wilting, slight root or stem rot, 3 = 50–75% strong wilting, pronounced rot, 4 = 75–100% leaf and stem death, complete plant collapse). Data obtained from bamboo samples were converted into percentage disease severity using the disease severity (DS) formula [11].

### 2.4. Identification of Pathogenic Fungal Isolates

#### 2.4.1. Macroscopic and Microscopic Identification of Pathogenic Fungal Isolates

The two fungal isolates (MF-1 and MF-2), identified as the most virulent as a result of the pathogenicity test, were grown on PDA, and the cultural and morphological characteristics of their colonies were examined. Lactophenol cotton blue, which specifically stains fungal structures, including chitin found in fungal cell walls, was used for the examinations, according to the reference studies by Leck [20] and Kim et al. [21]. Lactophenol cotton blue can specifically stain fungal structures, including chitin found in fungal cell walls [20,21]. The fungi were stained with lactophenol cotton blue and subjected to microscopic analysis using a Leica ICC50W microscope camera (Leica Microsystems GmbH, Wetzlar, Germany).

#### 2.4.2. Molecular Identification of Pathogenic Fungal Isolates

Fungi were incubated in PDA medium for 7 days. Fungal mycelia were collected from the incubated fungi using a loop, and genomic DNA was isolated using the PureLink^®^ Genomic DNA Mini Kit (Thermo Fisher Scientific, Waltham, MA, USA). according to the manufacturer’s instructions. The purity of the DNA obtained was assessed by running it on a 1% agarose gel at 75 V for 45 min.

Two genomic regions were targeted for molecular identification that includes the internal transcribed space (ITS) and translation elongation factor 1-α (TEF 1-α) regions [22]. The primers used for amplification were EF-1/EF-2 (F-CATCGAGAAGTTCGAGAAGG: R-TACTTGAAGGAACCCTTACC), targeting the nuclear translation elongation factor 1-alpha (tef1) gene; and ITS1/ITS4 (FCTTGGTCATTTAGAGGAAGTAA: R-TCCTCCGCTTATTGATATGC), targeting the internal transcribed spacer ITS-region [23]. Polymerase Chain Reaction (PCR) products were amplified under the specified conditions (95 °C for 2 min; 95 °C for 30 sec; 56 °C for 1 min; 72 °C for 1 min; 72 °C for 10 min; 35 cycles). DNA sequence analysis was performed for the amplified PCR sequence by a commercial service provider (BM Lab. Lab. Systems Ltd. Co., Ankara, Türkiye). The sequencing results were compared with ribosomal sequences in GenBank using the BLASTN 2.2.26+ program, and the sequence results were recorded in GenBank.

### 2.5. Purification of Chitinase Enzyme from Bacteria

The chitinase enzyme was partially purified by ammonium sulfate precipitation of bacteria. For this purpose, the bacteria homogenate was precipitated by ammonium sulfate precipitation at 0–100% ranges, and chitinase activity of the precipitate and the supernatant was examined to identify the range with the highest activity. The precipitate was dissolved in 0.1 M phosphate buffer (pH 7) and dialyzed against the same buffer [24].

### 2.6. Determining Chitinase Enzyme Activity

Chitinase enzyme activities were determined according to Senol et al. [16] and Dikbaş et al. [24]. The reaction was carried out by incubating 0.2 mL of enzyme solution with 0.5 mL of 0.5% (*w*/*v*) colloidal chitin (prepared in 50 mM citrate buffer, pH 6.0) at 37 °C for 30 min. Staining solutions were added to the reaction mixture and kept at 80 °C for 10 min, and activity was determined by monitoring the absorbance change at 540 nm against the blank sample. One unit (1 U) of enzyme activity was defined as the amount of enzyme that catalyzed the release of 1 µmol of N-acetylglucosamine per mL per minute under the specified assay conditions [25].

### 2.7. Determination of the In Vitro Antifungal Potential of Bacterial Isolates and Their Partially Purified Chitinase Enzymes

Antifungal studies were conducted on Petri dishes containing Potato Dextrose Agar (PDA). In all treatments, six mm diameter disks taken from pathogenic fungal cultures grown on PDA were placed in the center of the PDA-containing Petri dishes. In the bacterial treatment, biocontrol agents were collected with a sterile swab after 24 h of incubation in Petri dishes containing NA and seeded onto the edges of the dishes containing pathogenic disks. In the partially purified chitinase enzyme treatment, the enzyme was applied to the edges of the Petri dishes containing pathogenic fungal disks. In the control treatment, only Petri dishes containing pathogenic disks were used. Petri dishes were covered with parafilm and incubated at 24 °C for 7 days under a 15 h light/9 h dark photoperiod. In the control treatment, evaluations were made on the day the fungal growth occurred on the entire Petri dish, and the diameter of the developing colony of the pathogenic fungus was measured in millimeters. Each treatment was repeated three times using three Petri dishes under the same experimental conditions. Percentage inhibition rates were calculated by determining the mycelial growth (mm) diameters of fungal disks in the medium [15,26].

### 2.8. Characterization of Optimum and Stable Temperature and pH Conditions of the Chitinase Enzyme

Reactions were carried out at temperatures between 10 °C and 90 °C to determine the optimum enzyme activity temperature. For this purpose, the standard activity test method was used. To determine the optimum pH of chitinase enzyme, sodium acetate (pH 2–3), sodium citrate (pH 4.0–6.0), Tris–HCl (pH 7–9), and Na-carbonate (pH 10–11) buffers were used [27,28]. Colloidal chitin (0.5%) was used as a substrate in the standard activity test method to perform the measurements. The procedures for temperature stability and pH stability were performed based on the method described by Saribuga et al. [27]. For temperature stability, the enzyme solution was incubated at temperatures between 10 °C and 90 °C, and enzyme activity was measured every 15 min for 2 h. For pH stability, enzyme solutions adjusted to pH values between 2 and 11 were prepared, and hourly activity measurements were made for 9 h. Each study was performed in triplicate, and the data were averaged.

### 2.9. SDS-PAGE Electrophoresis

The enzyme’s purity was evaluated using the Laemmli [29] method via 3–8% gradient sodium dodecyl sulfate polyacrylamide gel electrophoresis (SDS-PAGE; Bio-Rad Laboratories, Hercules, CA, USA). Electrophoresis procedures were conducted in accordance with previously published protocols. Gels were visualized using the silver staining technique and imaged after the destaining process [30].

### 2.10. Protein Determination

Soluble protein concentration was measured using the Bradford [31] method by staining with Coomassie Brilliant Blue G-250 and bovine serum albumin as a standard.

### 2.11. Statistical Analysis

Data analysis was performed using analysis of variance (one way-ANOVA) in SPSS version 20.0 (SPSS Inc., Chicago, IL, USA), with mean differences assessed through Tukey’s HSD multiple range test. Statistical significance was set at a *p*-value of less than 0.05. All experiments were conducted five times, and the results are presented as the mean ± standard deviation with a 95% confidence interval [32].

## 3. Results

### 3.1. Isolation and Identification of Fusarium Isolates

**Figure 1:**
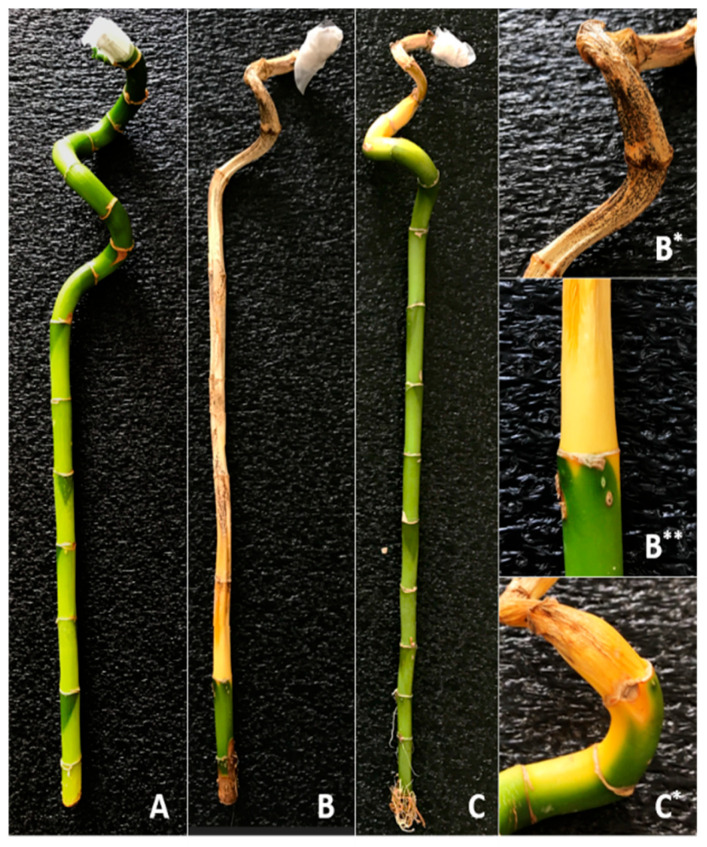
Image of *D. sanderiana* (lucky bamboo) virulence test after 15 days (**A**): Control (Only sterile water), (**B**): MF-1 application, (**C**): MF-2 application (B*), (B**): close-up of the symptom caused by MF-1, (C*): close-up of the symptom caused by MF-2.

In this study, five different pathogenic fungi were isolated to assess virulence factor and disease severity for the disease that causes brown-black necrotic spots on the stem, starting with yellowing of the leaves and progressing down the stem (top to bottom). Of the five fungal isolates, MF-1 and MF-2 exhibited pathogenic properties. In plants wounded at the apex, MF-1 and MF-2 isolates showed a virulence score of 3.8 and 1.6, respectively. The virulence score and disease severity results of MF-1 and MF-2 isolates on *Dracaena sanderiana* are shown in Table 1. Images of *D. sanderiana* (lucky bamboo) at the end of the pathogenicity experiment are shown in Figure 1.

Disease symptoms caused by isolates (MF-1 and MF-2) isolated from *D. sanderiana* include wilting starting from the top of the plant and progressing towards the root, causing yellowing of the leaves and stem. In addition, black spots appear on the stem of the plant in advanced stages of the disease (Figure 1).

**Figure 2:**
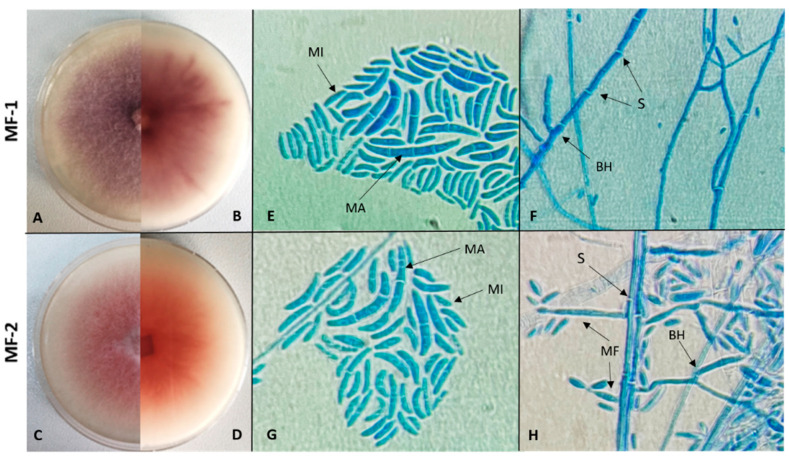
Morphological features, colony morphology, and microscopic characteristics of *Fusarium oxysporum* MF-1 and MF-2 isolated from lucky bamboo. Mycelial growth of *F. oxysporum* MF-1 (**A**,**B**) and MF-2 (**C**,**D**) on PDA medium after 10 days: (**A**,**C**)—top surface; (**B**,**D**)—reverse side of Petri dishes. Microscopic structures of MF-1 (**E**,**F**) and MF-2 (**G**,**H**), showing macroconidia (MA: large, multi-septate asexual spores), microconidia (MI: small, single-celled asexual spores), monophialides (MF: specialized spore-producing cells), branching hyphae (BH), and septa (S: cross-walls within hyphae). Magnification: 100× (objective lens).

Fungal colonies were obtained from single-spore isolation on potato dextrose agar (PDA). Colonies appeared white-lilac (MF-1), white-pink (MF-2), (Figure 2). The fungus was re-isolated only from diseased stems, fulfilling Koch’s postulates.

Isolates MF-1 and MF-2 showed similar characteristics upon microscopic examination. Microconidia of both were observed as unicellular, while macroconidia were sickle-shaped, slightly curved, and had 3–5 septate segments. Hyphal structures were also observed as septate and branched, but no chlamydospore structure was observed. According to sequence analysis of the internal transcribed spacer (ITS) region, the pathogenic fungal isolates MF-1 (GenBank accession no. ON863429) and MF-2 (GenBank accession no. ON862977) were identified as *Fusarium oxysporum*. Similarly, analysis of the translation elongation factor 1-alpha (TEF-1α) gene confirmed the identification of MF-1 (GenBank accession no. PX088141) and MF-2 (GenBank accession no. PX088142) *as Fusarium oxysporum*.

### 3.2. Partial Purification and Activity Profile of Chitinase Enzyme

**Figure 3:**
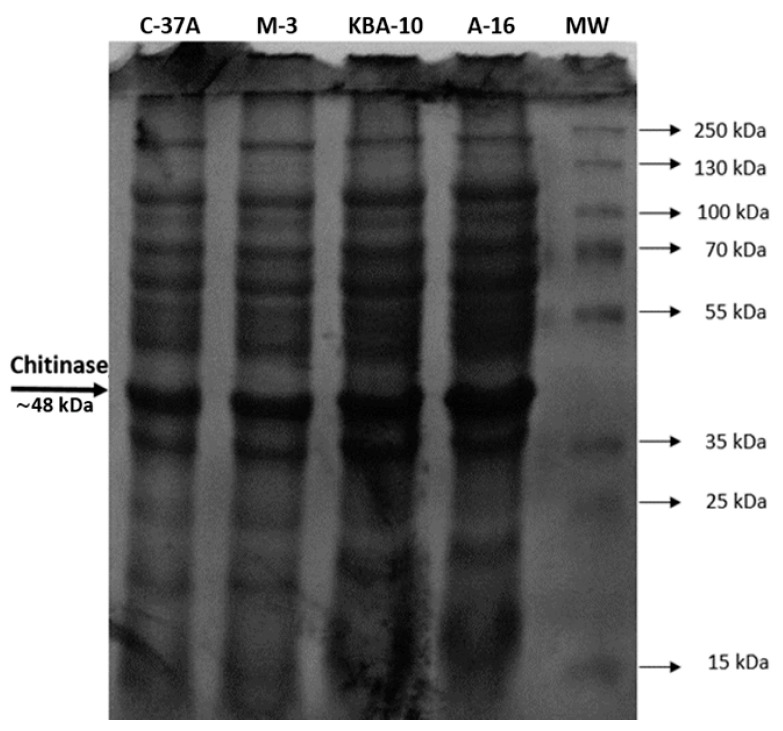
SDS–PAGE analysis image from bacteria and MW (marker molecular weight).

The purification results of four bioagent bacteria isolates and the ranges that yield the best enzyme activity are given in Table 2. The chitinase enzyme was purified from C-37A using 40–60% (NH_4_)_2_SO_4_ saturation with 1.01-fold purification and 87.16% recovery, from M-3 using 60–80% (NH_4_)_2_SO_4_ saturation with 0.9-fold purification and 19.61% recovery, from KBA-10 using 60–80% (NH_4_)_2_SO_4_ saturation with 0.82-fold purification and 10.0% recovery, and from A-16 using 60–80% (NH_4_)_2_SO_4_ saturation with 2.5-fold purification (Table 2). As a result of SDS–PAGE, chitinase had a molecular mass of approximately 48 kDa (Figure 3).

### 3.3. Optimum pH and Temperature Values of the Chitinase Enzyme

**Figure 4:**
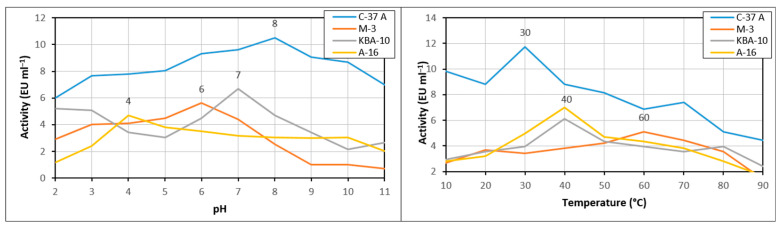
The effect of temperature and pH on purified chitinase enzyme activity from bioagent bacteria.

The enzyme activities of four strains were evaluated across a temperature range of 10 °C to 90 °C and pH levels 2 to 11. Among the tested isolates, C-37 A exhibited the highest chitinase activity at 30 °C, whereas M-3 reached its maximum activity at 60 °C. Both KBA-10 and A-16 showed peak activity at 40 °C (Figure 4). According to optimum pH results, C-37A exhibited the highest chitinase activity at pH 8, whereas M-3 reached its maximum chitinase activity at pH 6. KBA-10 and A-16 showed high activity, respectively, at pH 7 and pH 4 (Figure 4).

### 3.4. Stable Temperature and pH Values of the Chitinase Enzyme

**Figure 5:**
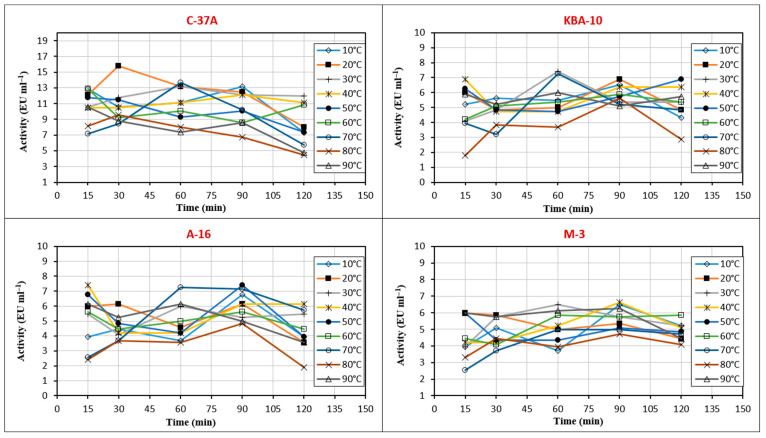
Stable temperature values of chitinase enzyme activity from bioagent bacteria.

In this study, the activity stability of chitinase enzymes of four different bacterial isolates (M-3, A-16, C-37A, and KBA-10) was evaluated at temperatures between 10 °C and 90 °C for 15 to 120 min (Figure 5). Stability values were evaluated according to increases and decreases, and balanced results were considered. Partially purified chitinase from C-37A showed stability at 30 and 40 °C for 120 min and retained 50% of its activity at 90 °C for 120 min. Partially purified chitinase from M-3 showed stability at 50 °C. Partially purified chitinase from A-16 showed stability at 40 °C for 120 min and retained 80% of its activity. Partially purified chitinase from KBA-10 showed stability between 10 °C and 40 °C for 120 min.

**Figure 6:**
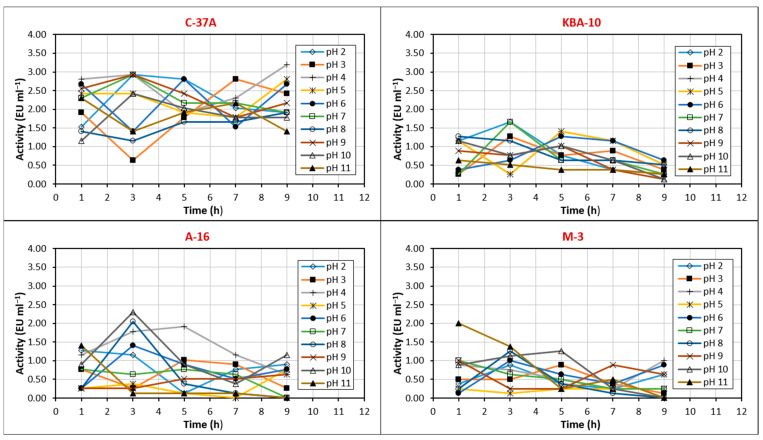
Stable pH values of chitinase enzyme activity from bioagent bacteria.

The study evaluated the activity stability of chitinase enzymes of four different bacterial isolates at pH between 2 °C and 11 °C for 1 to 9 h (Figure 6). Chitinase obtained from C-37A was stable at pH 7 and 8 for 3–7 h. The activity profile of chitinase from KBA-10 was irregular, with the most stable pH being pH 8, where 40% of enzyme activity was preserved. Chitinase from M-3 was stable at pH 5 for 9 h, and its activity did not decrease. Chitinase from A-16 showed high activity at pH 4 for the first 5 h and was stable at pH 7, maintaining 80% of its activity for 7 h.

Table 3 shows the fungal disk growth diameters and percent inhibition rates of suspensions of four bioagent bacterial isolates and partially purified chitinase enzymes tested in vitro for antifungal properties against pathogenic MF-1 and MF-2 fungi in Petri dishes. When the results of all groups were considered, they showed statistically significant differences compared to the control group.

**Figure 7:**
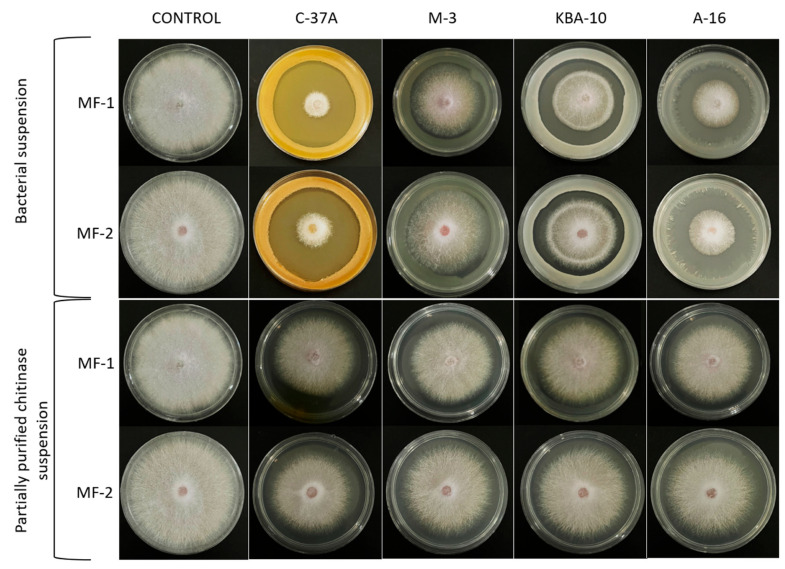
Inhibitory effects of four bioagent bacteria and purified chitinase enzyme on pathogenic fungus in PDA medium.

In the C-37A, fungal disk growth diameters of MF-1 and MF-2 fungi were determined as 14.2 and 18, respectively, while the percent inhibition rates were determined as 83.3% and 75.5%, respectively. In the A-16, M-3, and KBA-10 bioagents, the fungal disk growth diameters of MF-1 fungus were determined as 21.5, 32.2, and 40.5, respectively, and the fungal disk growth diameters of MF-2 fungus were determined as 25.3, 36.3, and 40.2, respectively. In the application of partially purified chitinase enzyme, the fungal disk growth diameter of MF-1 fungus was found to be approximately 41–44 mm, while the fungal disk growth diameter of MF-2 fungus was found to be approximately 43–44 mm. The inhibitory effects of the solutions of the four bioagent bacteria and the purified chitinase enzyme on MF-1 and MF-2 fungi are shown in Figure 7.

## 4. Discussion

Lucky bamboo (*Dracaena sanderiana*), a popular choice among ornamental plants used for indoor and outdoor decoration, is easy to care for and adaptable but susceptible to diseases. One of the most concerning fungi is *F. oxysporum*. While this fungus causes a variety of symptoms, the most significant symptom is reported to be *Fusarium* wilt [5,12]. The first visible symptom of *Fusarium* wilt begins with the gradual yellowing of the leaves. The leaves gradually wither, and the entire plant collapses [33,34]. In this study, lucky bamboos exhibiting symptoms similar to *Fusarium* wilt were obtained, and five isolates considered pathogenic were purified. Pathogenicity studies were conducted with five isolates, and disease severity and virulence factors were assessed. Two isolates (MF-1 and MF-2) were found to be pathogenic. On lucky bamboo, the disease severity caused by MF-1 was 90%, and that caused by MF-2 was 40%. No disease was observed in the control treatment or in the other isolates. The MF-1 isolate was found to have a significantly higher disease severity. Similar studies have reported similar results to those obtained in this study. In their pathogenicity study on the surface of lucky bamboo, Abdel-Rahman et al. [5] determined that *Colletotrichum gloeosporioides* had 97% disease severity, *F. oxysporum* 95%, and *Alternaria alternata* 91%. Another study reported that *C. gloeosporioides* had 100% disease severity on lucky bamboo [8]. In their study, Elshahawy et al. [10] determined the disease severities of *C. dracaenophilum* (isolate 1), *C. gloeosporioides* (isolate 2), *C. gloeosporioides* (isolate 3), and *Colletotrichum dracaenophilum* (isolate 4) isolates applied to the same plant as 11%, 13%, 11%, and 91%, respectively. Literature studies have reported similar results to those obtained in this study [5,8,10].

MF-1 and MF-2 isolates were evaluated according to morphological and molecular diagnostic results. In this study, isolates MF-1 and MF-2 showed sickle-shaped macroconidia with 3–5 septa, oval to cylindrical microconidia, and short monophialids. These findings exhibited the typical morphological features of *F. oxysporum* [35,36,37]. ITS and TEF-1α gene sequence analyses confirmed that the fungal isolates MF-1 and MF-2 were identified as *F. oxysporum.* Molecular diagnostics have become essential for the reliable identification and phylogenetic classification of *Fusarium* species. The internal transcribed spacer (ITS) region could serve as a universal barcode for identifying fungal species, while protein-coding genes such as translation elongation factor 1-alpha (TEF1-α) improved resolution at the species and form-specific levels [38]. The addition of molecular diagnostic techniques to morphological diagnosis has enabled the separation of pathogenic fungi much more efficiently.

Microbial chitinases are applied in various fields such as food production, pharmaceutical industries, and agriculture. Microorganisms capable of producing chitinase enzymes provide practical and environmentally friendly, sustainable biological effects against various phytopathogens, reducing and preventing the spread of diseases [17]. In this study, partial purification of chitinase enzyme from *P. chlororaphis* C37-A, *B. megaterium* M-3, *A. radiobacter* A-16, and *B. megaterium* KBA-10 isolates was performed using the ammonium sulfate precipitation method. The best precipitation range of partially purified chitinase from C-37A was determined to be 40–60% with an activity value of 10.83 EU/mL. Chitinase was purified 1.01-purification fold with a recovery of 87.16% and specific activity (EU/mg protein) of 2.43 from C-37A bacteria. According to the results, an increase in specific activity was observed after purification. At the same time, a decrease in recovery was detected, and the best purification coefficient was obtained in the C-37A and A-16 isolates. In similar studies, chitinase enzyme was obtained from Bacillus subtilis TV-125 with 0–20% (NH_4_)_2_SO_4_ saturation with 22.4-fold purification and 15.5% recovery [18], from *Paenibacillus sp.* TKU052 with 60% (NH_4_)_2_SO_4_ saturation with 3.17-fold purification and 45.95% recovery [39], and from *Stenotrophomonas maltophilia* with 30% (NH4)2SO4 saturation with 1.4-fold purification and 40.7% recovery [40].

Chitinases exhibit a broad molecular weight range of 20–90 kDa [41], while bacterial chitinases are generally within the ∼20–60 kDa range [42,43]. In the study, the molecular weight of chitinase was approximately 48 kDa by SDS-PAGE analysis. In other studies that support our findings, the molecular weight of chitinase enzyme was reported as 47 kDa for *Bacillus thuringiensis* [44], 52 kDa for *S. maltophilia* [40], 55 kDa for B. subtilis TD11 [45] and 70 kDa for *Paenibacillus* sp. TKU052 [39].

Enzymes are highly sensitive to environmental factors such as ionic strength, metal ions, and chemicals, as well as to parameters such as pH and temperature. Optimizing the temperature and pH stability of chitinase is crucial to maintain its activity and durability in a wide range of industrial applications, from biopesticide formulations to food processing and pharmaceutical industries [46]. The chitinase enzyme purified from the C-37A isolate showed optimum activity at 30 °C and pH 8. Furthermore, the enzyme was stable at pH 7 and 8 at 30–40 °C. This was in line with the optimum values. The purified enzyme lost only 50% of its activity at 90 °C at 120 min. It has a generally stable structure. The chitinase purified from the M-3 isolate reached optimum values at 60 °C and pH 6, and the enzyme was stable at pH 5 at 50 °C. The chitinase obtained from KBA-10 reached optimum values at 40 °C and pH 7. While the enzyme was stable between 10 °C and 40 °C, its stable pH profile was irregular, and its most stable value was determined to be pH 8. The chitinase obtained from A-16 showed optimum activity at 40 °C and pH 4 and was stable for 120 min at 40 °C, losing 20% of its activity. In addition, this enzyme showed high activity at pH 4 for 5 h and reached its most stable pH value at pH 7. The optimum and stable values of the enzyme obtained from A-16 were also found to be parallel. According to these results, the chitinases of C-37A and KBA-10 showed good activity and stability in neutral and slightly basic environments, while the chitinases of A-16 and M-3 showed good activity and stability in more acidic environments. While the chitinase obtained from M-3 had a thermophilic enzyme structure at 50–60 °C, the others showed stability at approximately 30–40 °C. In a study, the optimum pH and temperature values of purified chitinase of *Bacillus* sp. R2 are 7.5 and 40 °C, respectively. The enzyme showed stability at 40 °C and pH between 7 and 8 for one hour [47]. This study is similar to the results obtained, especially for isolate C-37A. According to Chaiharn et al. [44], the optimal activity of the chitinase of *B. thuringiensis* R 176 was pH 7 and 37 °C. More than 80% of R 176 was stable at pH 6 to 8 and more than 90% at 40 °C. The optimum temperature of chitinase obtained from *Paenibacillus* sp. A1 was 50 °C and pH 4.5. It was also determined that this chitinase was stable at 45 °C and pH 4.5–5.5 [48]. Other studies have reported that the optimum temperatures of most bacterial chitinases are generally in the range of 40–60 °C and pH 4–10. These studies support the results obtained in our study [24,39,40,48,49].

Bioagent bacteria and their metabolites are promising biocontrol agents in the control of diseases caused by *Fusarium* species [50]. For this reason, in this study, the antifungal activity of four biomagnifying bacteria suspensions and partially purified chitinase enzymes were evaluated in vitro. C-37A isolate significantly inhibited the growth of both pathogenic fungi and the percentage inhibition rates of MF-1 and MF-2 pathogens were approximately 83% and 75%, respectively. In addition, the percent inhibition of pathogenic fungi by other isolates ranged from 68 to 29%, with *A. radiobacter* A-16, *B. megaterium* M-3, and *B. megaterium* KBA-10 ranging from the highest to the lowest, respectively. The percentage inhibition of the chitinase enzyme isolated from the bioagents was 28% for C-37A, 24% for KBA-10 and M-3, and 22% for A-16 isolate. The percentage inhibition rates of the partially purified chitinase enzyme for MF-2 isolate were in the same group statistically for all bioagents and were in the range of 23–21%. *P. chlororaphis* C-37A isolate, which exhibits high chitinase activity, showed the highest percentage inhibition rate for isolates MF-1 and MF-2 in both bacterial suspension and partially purified enzyme solution. KBA-10 and M-3, two *B. megaterium* isolates with similar specific activity after partial purification, also inhibited pathogenic fungi with the same (MF-1) or very similar (MF-2) percentage inhibition rates. The study findings demonstrate the importance of chitinase in antifungal activity. A previous study supports the antifungal activity of chitinase from different bacteria. The antifungal activity of *P. chlororaphis* (MF-1 and C37-A) and B. subtilis (TV-6F and TV-17C) isolates against the fungus *F. oxysporum* f.sp. *radicis-cucumerinum* ET 46 was investigated, and the percentage inhibition rate was measured as 35.2%, 34.1%, 31.7% and 31.7% for C-37A, MF-1, TV-17C, and TV-6F, respectively [15]. Another study showed that the chitinase purified from *Bacillus subtilis* NPU 001 strain inhibited the hypha extension of *F. oxysporum*; the chitinase enzyme partially purified from *Bacillus subtilis* TV-125A was highly effective against pathogenic *Fusarium culmorum* [15,51]. Chitinase from *Bacillus cereus* had antifungal activity against *Rhizoctonia. solani* and *F. oxysporum* [52], and chitinase enzyme obtained from *Bacillus pumilus* SG2 strongly inhibited the development of *Fusarium graminearum* [53]. The purified chitinase from *S. maltophilia* was used as a biocontrol agent against the fungus *F. oxysporum* [40]. In this study, it was determined that bioagent bacteria and the obtained chitinase showed antifungal activity against *F. oxysporum* MF-1 and *F. oxysporum* MF-2 in in vitro tests and inhibited the growth of phytopathogenic fungi.

## 5. Conclusions

In this study, *F. oxysporium* MF-1 and *F. oxysporium* MF-2 were isolated and identified from lucky bamboo (*D. Sanderiana*) in Türkiye. Four different bacteria and partially purified chitinase enzymes from these bacteria were used for biocontrol to limit *F. oxysporium*, causing *Fusarium* wilt in lucky bamboo, and positive results were obtained. Among the biocontrol bacteria, isolates C-37A and A-16, which showed the highest chitinase activity, were also found to have the best antifungal activity. In addition, chitinase purified from C-37A maintained its stability in basic medium and at high temperature, while chitinase obtained from A-16 showed high stability in acidic medium at 40 °C. In conclusion, considering the findings obtained, it is concluded that the bioagents offer sustainable biological effects against *F. oxysporium* in lucky bamboo and that the chitinase enzyme purified from these isolates can be used primarily as a biocontrol agent in agriculture as well as potentially in various industrial applications. In vivo studies are also needed to fully evaluate the efficacy of the bioagents.

## Figures and Tables

**Table 1:** Virulence score and disease severity values of fungal isolates on *Dracaena sanderiana*.

Application	Virulence Score (0–4)	Disease Severity (%)
MF-1	3.8 ± 0.4	90 ± 12.2
MF-2	1.6 ± 0.5	40 ± 10.0
Control	0 ± 0	0 ± 0

0 = no disease; 1 = 1–25% slight wilting and yellowing, 2 = 26–50% pronounced wilting, slight root or stem rot, 3 = 50–75% strong wilting, pronounced rot, 4 = 75–100% leaf and stem death, complete plant collapse.

**Table 2:** The purification steps for chitinase from bacteria.

Bacteria	Purification Step	Volume (mL)	Activity (EU/mL)	Protein (mg/mL)	Total Activity (EU/mL)	Total Protein (mg)	Specific Activity (EU/mg)	Recovery %	Purification Fold
	Crude Extract	40	10.83	4.53	433.20	181.40	2.39	100	1
C-37A	Ammonium Sulfate Precipitation (40–60%)	40	9.44	3.87	377.60	154.8	2.43	87.16	1.01
	Crude Extract	40	2.55	1.87	102	74.80	1.36	100	1
M-3	Ammonium Sulfate Precipitation (60–80%)	40	0.50	0.41	20	16.40	1.22	19.61	0.90
	Crude Extract	40	5.10	2.85	204	114	1.78	100	1
KBA-10	Ammonium Sulfate Precipitation (0–20%)	40	0.51	0.35	20.40	14	1.46	10	0.82
	Crude Extract	40	2.81	1.06	44.96	42.40	1.06	100	1
A-16	Ammonium Sulfate Precipitation (60–80%)	40	1.02	0.39	40.8	15.39	2.65	90.74	2.5

**Table 3:** The effect of bioagent bacteria and their partial purification chitinase on the growth of pathogenic fungus.

Bacteria		MF-1		MF-2	
		FDGD (mm)	PIR (%)	FDGD (mm)	PIR (%)
Bacterial isolates	C-37A	14.2 ± 0.2 ^a^	83.3 ± 0.5 ^a^	18.0 ± 0.7 ^a^	75.5 ± 1.4 ^a^
	M-3	40.5 ± 1.1 ^d^	29.3 ± 2.2 ^d^	40.2 ± 0.2 ^d^	30.3 ± 0.5 ^d^
	KBA-10	32.2 ± 0.6 ^c^	46.4 ± 1.3 ^c^	36.3 ± 1.0 ^c^	38.1 ± 2.1 ^c^
	A-16	21.5 ± 0.4 ^b^	68.3 ± 0.8 ^b^	25.3 ± 1.3 ^b^	60.5 ±2.6 ^b^
	Control	54.8 ± 0.2 ^e^	0.0 ± 0.5 ^e^	55.0 ± 0.0 ^e^	0.0 ± 0.0 ^e^
	F-Values *	1390.8	1384.1	640.2	638.0
Partial purification enzyme solution	C-37A	41.2 ± 2.0 ^a^	28.0 ± 4.2 ^a^	43.7 ± 1.0 ^a^	23.1 ± 2.1 ^a^
	M-3	42.8 ± 0.2 ^ab^	24.6 ± 0.5 ^ab^	44.3 ± 1.3 ^a^	21.7 ± 2.6 ^a^
	KBA-10	42.8 ± 0.5 ^ab^	24.6 ± 1.0 ^ab^	44.7 ± 0.5 ^a^	21.1 ± 0.9 ^a^
	A-16	44.0 ± 0.4 ^b^	22.2 ± 0.8 ^b^	44.0 ± 0.8 ^a^	22.5 ± 1.7 ^a^
	Control	54.8 ± 0.2 ^c^	0.0 ± 0.5 ^c^	55.0 ± 0.0 ^b^	0.0 ± 0.0 ^b^
	F-Values *	64.4	64.6	67.5	67.8

Note: Values in the table are given as (mean  ±  standard deviation). The numbers in one column having the same letter are not significantly different. PIR: Percent inhibition rate of the bioagent bacteria, FDGD: Fungal disk growth diameter. *: Values belong to ANOVA.

## Data Availability

The original contributions presented in this study are included in the article. Further inquiries can be directed to the corresponding author.
